# Association between Physician-Diagnosed Asthma and Weight Status among Chinese Children: The Roles of Lifestyle Factors

**DOI:** 10.3390/ijerph17051599

**Published:** 2020-03-02

**Authors:** Lijuan Lai, Ting Zhang, Xia Zeng, Weiqing Tan, Li Cai, Yajun Chen

**Affiliations:** 1 Department of Maternal and Child Health, School of Public Health, Sun Yat-sen University, Guangzhou 510080, China; lailj@mail2.sysu.edu.cn (L.L.); zengx26@mail2.sysu.edu.cn (X.Z.); 2 School of Public Health, Li Ka Shing Faculty of Medicine, The University of Hong Kong, Hong Kong, China; zhangt77@hku.hk; 3 Health Promotion Centre for Primary and Secondary Schools of Guangzhou Municipality, Guangzhou 510020, China; twq13560033191@163.com

**Keywords:** asthma, pediatric obesity, lifestyle, screen time

## Abstract

Childhood asthma and obesity have posed a parallel epidemic over the past few decades. However, whether asthma diagnosis is associated with obesity, and what the roles of lifestyle factors play in this relationship, remained unclarified. This study aimed to investigate the association between asthma and weight status in Chinese children and explore the potential mediating and/or modifying roles of lifestyle factors in the association. In this cross-sectional study, 16,837 children aged 6–12 years were recruited from Guangzhou, China. Participants’ information on physician-diagnosed asthma was collected from parents, and data on physical activity, screen time, and sleeping were reported in a validated questionnaire. Height and weight were objectively measured, and weight status was classified by body mass index (BMI). Multiple logistic regression analysis and mediation analysis were used. Results showed that asthmatic children were at significantly higher risk of obesity (odds ratio (*OR*) 1.51, 95% confidence interval (*CI*) 1.03, 2.21) compared with non-asthmatic children. More importantly, this increased risk was even greater in children with moderate-to-vigorous physical activity <60 min/d and children with screen time >2 h/d (both *P*_interaction_ < 0.05). Also, a positive relationship of asthma with overweight was found in children with screen time >2 h/d (*OR* 3.92, 95% *CI* 1.56, 9.88), while a negative association was observed between asthma and underweight in children aged 9–12 years (*OR* 0.23, 95% *CI* 0.06, 0.92). Mediation analysis indicated that these associations were not mediated by physical activity, screen time, or sleeping. The findings suggested that physician-diagnosed asthma was associated with higher risks of overweight and obesity, and these risks might be exacerbated by insufficient physical activity and prolonged screen time.

## 1. Introduction

Asthma and obesity remained public health problems of global concerns in children, and have emerged in a parallel increase in prevalence worldwide over the past few decades [1]. Numerous studies have long focused on the unidirectional association from obesity to asthma, and repeatedly documented that obesity increases the risk of asthma, worsens asthma morbidity, and decreases response to drugs [2,3]. However, whether asthma leads to a higher risk of obesity remains uncertain. Some cross-sectional studies suggested that children with asthma had higher body mass index (BMI) and higher prevalence of obesity than those without [4,5,6,7], whilst others reported no significant difference in BMI [8] or overweight/obesity prevalence [9]. The few longitudinal studies showed that asthma was associated with higher risks of subsequent weight gain and/or obesity in children [10,11,12]. In contrast, results from other studies did not support the effect of asthma on adiposity accumulation [13] or overweight/obesity development [14]. Additionally, it is worth noting that the vast majority of existing evidence was based on data from the US [7,8,10,11] or European countries [5,6,12], where the prevalence of asthma and obesity are relatively high [15,16], conferring an uncertainty when applied to other populations. To our knowledge, only one prior study has examined the effect of asthma on adiposity among 5138 Chinese-ancestry children aged 9–13 years [13]. Given that ethnic differences do exist in the association between asthma and obesity [17], there is a key research gap in the literature. If asthma does increase the risk for obesity, it is of concern for clinicians to monitor BMI and control weight in asthmatic children to prevent comorbidity of asthma and obesity.

It is well-established that obesity is an acquired health condition occurring as a consequence of energy imbalance and could be influenced by lifestyle factors [18], with no exception among children with asthma [19]. Thus, one potential mechanism proposed to link asthma to obesity risk was through changes in weight-related lifestyle behaviors. As previously reported, asthmatic children were more likely to have an undesirable lifestyle, such as reduced physical activity (PA), prolonged screen time, and poor sleep hygiene, than their non-asthmatic peers [20]. These weight-related behaviors have long been appraised as contributors to the increased risk of obesity [21]. Also, these unhealthy lifestyle factors were reported to be associated with worse respiratory symptoms [22] and higher inflammatory stimulus [23], two factors identified as hazardous to both asthma and obesity. Given this, it is likely that lifestyle behaviors may mediate or bias the relationship between asthma and obesity. However, existing studies have rarely included these lifestyle factors into the analysis [11,12], leading to confusing findings and little knowledge about the potential roles of lifestyle behaviors in the relationship between asthma and obesity. 

Given the increasing prevalence of both asthma [24] and obesity [25], as well as the large population of Chinese children, it is imperatively vital to examine the relationship between asthma and obesity in this population to prevent comorbidity of the two conditions. A comprehensive understanding of the potential roles of lifestyle factors would help in clarifying the association between asthma and weight status and driving the development of specific and effective preventive strategies for asthma-complicated obesity. Therefore, this study aimed to: (1) evaluate the relationship of asthma with weight status in a large sample of Chinese children, and (2) explore the potential mediating and/or modifying role of lifestyle factors (i.e., PA, screen time, sleeping) in the association. We hypothesized that children with asthma were more likely to be overweight or obese than those without, and these risks might be explained and/or modified by different lifestyle factors.

## 2. Methods

### 2.1. Study Design and Participants

This cross-sectional study was conducted in Guangzhou, one of the largest cities in southern China, between September 2015 and June 2016. Using a random cluster sampling method, 13 primary schools were selected from 11 districts in Guangzhou. With the principals’ agreement, all students in grades 1–6 were invited to participate in the study. Originally, 22,816 students and their parents were recruited, and the sample size fell to 16,837 after excluding students with an age younger than 6 years or older than 12 years (*n* = 591), abnormal physical conditions (e.g., having cardiovascular, respiratory or endocrine diseases, *n* = 29), and those with missing data on BMI (*n* = 25) or asthma diagnosis information (*n* = 5334). The study was conducted in accordance with the Declaration of Helsinki, and the protocol was approved by the Ethical Review Committee for Biomedical Research, Sun Yat-sen University. All subjects and their parents gave informed consent for inclusion before they participated in the study.

### 2.2. Data Collection

Physician-diagnosed asthma was assessed by asking parents “Has the child been diagnosed with asthma by a physician?” through a written questionnaire. If the response was “yes”, the child would be considered as asthmatic. Parents also reported socio-demographic characteristics, including paternal and maternal education level (both categorized as “primary school or below”, “junior high school”, “senior high school”, “junior college”, and “college or above”) and monthly family income (categorized as “0~4999 Renminbi (RMB, also named Chinese Yuan)” (equivalent to 0~720 USD), “5000~7999 RMB ” (equivalent to 721~1152 USD), “≥8000 RMB” (equivalent to ≥1153 USD), and “refuse to disclose”) by the questionnaire.

Children’s lifestyle factors, including physical activities, sedentary behavior, and sleeping, were evaluated by children together with their parents using a questionnaire. To determine the habitual PA, the International Physical Activity Questionnaire-Short Form (IPAQ-SF) was used to collect the child’s frequency (days) and duration (minutes) of vigorous physical activities (e.g., running and basketball) and moderate physical activities (e.g., cycling and badminton) in the last 7 days, respectively. Vigorous PA (VPA, min/day) and moderate PA (MPA, min/day) were continuous variables which could be calculated directly from the questionnaire. Moderate-to-vigorous PA (MVPA) was the sum of MPA and VPA. Similarly, children also reported daily duration in minutes of doing homework, viewing television, and using computers after school in the past 7 days. Total sedentary time was calculated as the sum of the time spent on the aforementioned sedentary behaviors. Screen time was calculated by summarizing the daily time of viewing television and using computers. For sleep duration, children reported usual bedtime, wake-up time, sleep latency (minutes), and sleep duration at noon (minutes) in the past 7 days. Daily night sleep duration was assessed by calculating the period between bedtime and wake-up time subtracted by sleep latency. Also, food intake was assessed by asking the frequency (days) and amount (servings) of fruit, vegetable, meat products, and soft drink consumption, and the frequency (times) of fried food consumption (e.g., fried chicken and potatoes). Average daily intake of fruit, vegetable, meat products, and soft drink was calculated by the formula: average daily intake = (days × (amount in each of those days))/7. The questionnaires had been revised and piloted before the study and were found to have acceptable reliability and validity [26].

Children’s height (cm) and weight (kg) were measured by trained technicians according to standardized procedures based on students’ routine physical examination. Height was measured using a portable stadiometer, and weight was measured using a lever scale with students wearing light clothes and no shoes. Body mass index (BMI) was derived by dividing weight (kg) by height squared (m^2^). Chinese definition was adopted to classify underweight [27], overweight, and obesity [28] in children. For the reason that the Chinese criteria for overweight did not include the cutoffs for children aged 6 years, the World Health Organization (WHO) standard [29] was used to define overweight for this population. In addition, BMI z-score in this study was also calculated using WHO standards. 

### 2.3. Statistical Analysis

Differences of characteristics and lifestyle factors between children with and without physician-diagnosed asthma were evaluated using Pearson Chi-square tests and an independent *t*-test for categorical and continuous variables, respectively. Multilevel logistic regression analyses were performed to evaluate the associations of physician-diagnosed asthma with underweight, overweight or obesity (versus normal-weight), with adjustment for sex, age, paternal and maternal education levels, household income, food intake, MVPA, screen time, and night sleep. 

To explore whether effect modification exists, we further included interaction terms in the logistic regression model to test the interactions of asthma with gender, age, and lifestyle factors (i.e., MVPA, screen time, and night sleep duration). We also stratified the study participants by gender, age, and lifestyle factors, and investigated the association between asthma and weight status in each mentioned subgroup. According to whether they adhered to the recommendations for MVPA (≥60 min/d), screen time (≤2 h/d), or night sleep duration (≥9 h/d), participants were classified into two levels, respectively.

In order to explore whether lifestyle factors mediated the association between physician-diagnosed asthma diagnosis and weight status (normal weight versus underweight, overweight, or obesity), mediation analyses were conducted using the Karlson Holm Breen-command [30]. Each potential mediator was included in the model separately with adjustment of the aforementioned sociodemographic covariates. All statistical analyses were performed using Stata version 15.0. A two-tailed *p* < 0.05 was considered statistically significant in all analyses. 

## 3. Results

**Figure 1:**
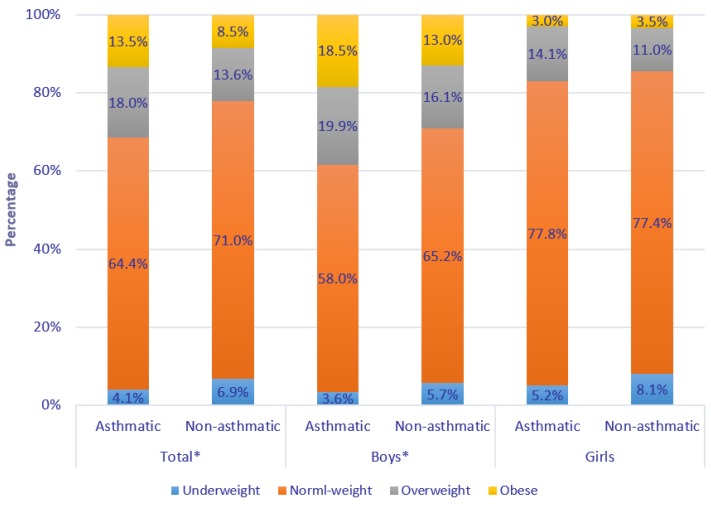
Distribution of weight status in children with or without physician-diagnosed asthma. Note: Difference of weight status between children with and without physician-diagnosed asthma was evaluated using Chi-square tests; * *p* < 0.05.

Among the 16,837 children aged 6–12 years (mean 8.67 ± 1.70 years), 416 (2.5%) children were reported to have physician-diagnosed asthma by their parents. Children with physician-diagnosed asthma were more likely to be boys, and their father and mother were more likely to have higher education (all *p* < 0.05) (Table 1). Overall, asthmatic children had significantly higher BMI and were more likely to be overweight or obese than non-asthmatic ones (18.0% and 13.5% versus 13.6% and 8.5%, *p* < 0.05), and similar results were observed in boys (Figure 1).

Compared with non-asthmatic children, asthmatic children were less likely to achieve the recommended ≥60 min/d of MVPA (30.9% versus 37.3%) and ≥9 h/d of night sleep (40.6% versus 46.4%), and had less time spent in vigorous PA and shorter total sleep duration (all *p* < 0.05). There were no significant differences in sedentary behavior or screen time between asthmatic and non-asthmatic children (Table 2). 

As shown in Table 3, in general, physician-diagnosed asthma was associated with 51% higher risk of obesity (odds ratio (*OR*) 1.51, 95% confidence interval (*CI*) 1.03, 2.21) than their non-asthmatic peers, with adjustment for gender, age, parental educational levels, household income, food intake, MVPA, screen time, and night sleep duration. There existed statistically significant interactions between asthma and age group, MVPA level, and screen time level (all *P*_interaction_ < 0.05). Specifically, subgroup analyses showed a significant negative association between asthma and underweight in children aged 9–12 years. While for asthma with overweight, a stronger association was observed in those with screen time > 2 h/d. Likewise, the magnitude of the risk for obesity due to asthma was larger in children with MVPA < 60 min/d and children with screen time > 2 h/d. 

As for mediation analysis, we did not observe any significant indirect effect of lifestyle factors in the association between asthma and weight status (Table 4).

## 4. Discussion

In this large general sample of Chinese children, we found that physician-diagnosed asthma was associated with a 51% higher risk of obesity than their non-asthmatic peers, with adjustment for socioeconomic indicators and reported lifestyle factors. The magnitude of the association was even larger in children with MVPA <60 min/d and children with screen time >2 h/d, indicating that insufficient MVPA and prolonged screen time might exacerbate the risk of overweight and obesity related to asthma. These results might be of benefit to advance specific and effective preventive strategies for asthma-complicated obesity.

Although it was most widely accepted that obesity or its metabolic complications constitute a risk factor for asthma and asthma morbidity, epidemiological evidence is accumulating on the reverse causal association from asthma to subsequent obesity. In addition, a recent study has identified co-expression network modules related to incident obesity among asthmatic children [31], providing biological evidence on asthma preceding the development of obesity. Coinciding with two population-based studies conducted on US children [4,7], the present results demonstrated that asthmatic children were at significantly increased risk of overweight and obesity, extending the literature with evidence from a large sample of Chinese children. These findings highlighted the imperative to monitor BMI and control weight routinely among the asthmatic children, even in regions exhibiting a relatively low prevalence of asthma and obesity, to combat comorbidity of asthma and obesity. However, it is worth noting that the only longitudinal study [13] in China investigating the causal relationship between asthma and adiposity only showed a small effect of asthma on adiposity accumulation among children aged 9–13 years, which is inadequate to clarify the underlying causality. Thus, more longitudinal data from diverse populations will be necessary to elucidate this conundrum. 

A previous systematic review of observational studies investigating the relationship between asthma and overweight in youth suggested that children with asthma had reduced PA and higher BMI compared to non-asthmatic peers, thus hypothesizing that PA likely mediates the relationship between asthma and overweight [32]. They believed that if children with asthma face barriers to regular PA, it is plausible that PA is a mediating factor in the development of obesity in asthmatic youth. However, to our best knowledge, this hypothesis has never been examined directly. Indeed, owing to fear or concern about the health condition [33], asthmatic children were more likely to have lower levels of PA, especially in those with severe atopic symptoms [34] or poor asthma control [35], compared to their non-asthmatic counterparts. In the current study, we found that asthmatic children exhibited a lower level of VPA and were less likely to achieve the PA recommendation, compared with their non-asthmatic counterparts, which was aligned with some prior studies [36,37]. Nevertheless, our results demonstrated little evidence on the mediating effect of PA, showing that the elevated risk of overweight and obesity in asthmatic children seemed not to be explained by their reduced PA. Interestingly, a positive association between asthma and obesity was observed only in children who did not comply with the recommendation of 60 min/d in MVPA, but not in those reached. This might be partially due to the positive association between physical inactivity and severe atopic disease [34], as well as low fitness [5,38], which significantly elevates the obesity risk among children with asthma. On the contrary, increasing levels of PA have a protective effect against the development of both asthma and obesity later in childhood [39]. Accordingly, the knowledge may be beneficial for tailoring daily-activity interventions for those physically inactive children with asthma to improve weight status.

Nowadays, as “digital natives”, children have grown up surrounded by digital information and entertainment on screens, which was evidenced as harmful to health profiles [40]. Prolonged screen time has been reported to increase the risk of both asthma [41] and weight gain, while few studies have examined the role of it when exploring an asthma–obesity association. Some studies revealed that children may use screen time for relaxation or as an alternative to PA due to their asthma symptoms [42], especially in those with severe asthma [34]. Therefore, they seemed more sedentary than non-asthmatic peers, which might contribute to their increased inactivity and subsequent obesity development [20]. In contrast, others demonstrated comparable levels of sedentary activities [6,43] and screen time [44,45] between children with and without asthma. Our results showed that screen time in asthmatic children did not differ from that in non-asthmatic children, nor did screen time explain the increased risk of overweight and obesity in relation to asthma. It is worth mentioning that the magnitudes of the associations of asthma with overweight and obesity were significantly increased in children with daily screen time over 2 hours, indicating that screen time might exacerbate the detrimental effects of asthma on overweight and obesity. This might be explained by the high energy intake [46] and worse asthma symptoms [47] linked to screen-based behaviors, such as television viewing, which predict increased risk of obesity. Additionally, asthma and screen time had independent influences on obesity [7], so that asthma in combination with excess screen time might entail a much higher risk of obesity than that separately. Hence, children with prolonged screen time may be more susceptible to the obesogenic effects of asthma and need more attention. Although it is challenging, future efforts should be made to reduce the asthmatic children’s time spent on screen-based behaviors, such as television viewing, computer, cellphone, and so on.

Aside from lifestyle factors, there posited several potential mechanisms linking asthma with increased obesity risk. One is medication use, including corticosteroids and antidepressant medications. Some studies showed that long-term treatment with corticosteroids, even in inhaled forms [48], can influence lipid metabolism by increasing the uptake of lipids from the digestive system and enhancing lipids’ storage in tissues, especially in the trunk [49]. Additionally, depressive symptoms were common and associated with asthma activity in children [50], and the use of some antidepressant medications can also lead to weight gain and be responsible for some proportion of the excess obesity [51]. The second biological mechanism was low fitness. Asthmatic children seemed less physically fit than non-asthmatics, which could not be accounted for by their bronchoconstriction [5]. Over the long term, a lack of physical fitness could predispose one to weight gain [38]. Additionally, systemic inflammation including adipokines [52], as well as common exposures that predispose individuals to both of these conditions [53], have also been reported to be underlying factors for the increased risk of obesity due to asthma. However, evidence on these hypotheses is limited. Given that obese patients tend to have worse asthma control and increased hospitalizations and do not respond to standard controller therapy as well as lean patients with asthma [54], more longitudinal studies are needed to better understand the underlying mechanisms (not merely through lifestyle behaviors) and to tackle these intractable concerns.

This study was strengthened by a large representative sample of Chinese children in one of the largest cities in China. This is also the first study to comprehensively investigate the potential roles of lifestyle factors in the association between asthma and weight status. However, several limitations warranted consideration in interpreting these results. Firstly, as a cross-sectional study, the presence of obesity and asthma are determined simultaneously. Thus, we were limited in our ability to ascertain the temporal relationship between asthma and obesity and could not rule out the possibility of reverse causation. Secondly, information on physician-diagnosed asthma and lifestyle factors was collected through self-reported questionnaires, which might introduce measurement error and potential recall bias. However, such measurement error would likely lead to a non-differential bias, potentially leading to underestimating the true effects. Thirdly, due to a lack of information on clinical presentation, severity, treatment (especially corticosteroids use), and pathobiology of asthma, we did not manage to access the disease heterogeneity in all observed asthma individuals. Finally, although we have adjusted for demographic confounders in the models, the possibility of residual confounding by unmeasured factors, including puberty and the comorbidity of asthma (i.e., obstructive sleep apnea), limited the interpretation of these findings and may explain inconsistencies across studies.

## 5. Conclusions

Physician-diagnosed asthma was associated with increased risks of overweight and obesity among a large sample of Chinese children. These risks might be exacerbated by insufficient physical activity and prolonged screen time. Lifestyle factors, such as PA, screen time, and sleeping, did not explain the association between asthma and weight status.

## Figures and Tables

**Table 1:** Characteristics of the total sample (*n* = 16,837).

Characteristics	Total	Asthmatic Children	Non-Asthmatic Children	*p* *
*n* (%)	16,837 (100.0)	416 (2.5)	16,421 (97.5)	*-*
Boys (%)	8878 (52.7)	281 (67.5)	8597 (52.4)	<0.001
Age group				0.913
6–8 years	7937 (47.1)	195 (46.9)	7742 (47.1)	
9–12 years	8900 (52.9)	221 (53.1)	8679 (52.9)	
Paternal education level (%)				<0.001
Junior high school or below	1948 (11.9)	29 (7.1)	1919 (12.0)	
Senior high school	7469 (45.6)	148 (36.3)	7321 (45.8)	
College or above	6965 (42.5)	231 (56.6)	6734 (42.2)	
Maternal education level (%)				<0.001
Junior high school or below	2516 (15.3)	25 (6.2)	2491 (15.6)	
Senior high school	7864 (48.0)	173 (42.7)	7691 (48.1)	
College or above	6011 (36.7)	207 (51.1)	5804 (36.3)	
Monthly household income (%)				0.083
0~4999 RMB	3337 (20.3)	67 (16.5)	3270 (20.4)	
5000~7999 RMB	3436 (20.9)	81 (20.0)	3355 (20.9)	
≥8000 RMB	6836 (41.5)	192 (47.3)	6644 (41.4)	
Refuse to disclose	2851 (17.3)	66 (16.3)	2785 (17.3)	
Height (cm)	135.6 ± 11.7	136.2 ± 11.4	135.6 ± 11.7	0.313
Weight (kg)	31.3 ± 9.9	32.9 ± 10.9	31.2 ± 9.9	0.002
BMI (kg/m^2^)	16.6 ± 3.1	17.3 ± 3.5	16.6 ± 3.1	<0.001
BMI z-score ^#^	−0.1 ± 1.4	0.2 ± 1.5	−0.1 ± 1.4	<0.001

RMB, Renminbi (A common unit of money in China); BMI, Body mass index. Note: Data are presented as Mean ± standard deviation (SD) for continuous variables and percentages for categorical variables; * *p*-value denotes independent *t*-tests for continuous variables and Pearson Chi-square tests for categorical variables between children with and without asthma; ^#^ BMI z-score was calculated by age- and sex-specific BMI cutoffs proposed by the World Health Organization (WHO) inference 2007.

**Table 2:** Differences in lifestyle factors between children with and without physician-diagnosed asthma.

Behavior Items	Total (*n* = 16,837)			Girls (*n* = 7959)			Boys (*n* = 8878)		
	Asthmatic	Non-Asthmatic	*p* *	Asthmatic	Non-Asthmatic	*p* *	Asthmatic	Non-Asthmatic	*p* *
**Physical activity**									
MVPA ≥ 60 min (%)	107 (30.9)	5022 (37.3)	0.015	29 (25.9)	2239 (34.6)	0.055	78 (33.3)	2783 (39.9)	0.045
MVPA (min/d)	53.6 ± 46.3	57.6 ± 48.4	0.115	50.9 ± 46.5	54.9 ± 46.7	0.372	54.9 ± 46.3	60.1 ± 49.9	0.092
VPA (min/d)	25.6 ± 27.0	28.6 ± 28.8	0.038	23.0 ± 24.0	25.7 ± 26.8	0.294	26.8 ± 28.3	31.3 ± 30.3	0.015
MPA (min/d)	28.7 ± 29.9	29.3 ± 29.7	0.715	28.2 ± 29.9	29.2 ± 29.2	0.708	28.9 ± 29.9	29.3 ± 30.1	0.848
**Sedentary behavior**									
Total sedentary time (h/d)	2.8 ± 1.4	2.9 ± 1.3	0.457	2.7 ± 1.4	2.8 ± 1.3	0.400	2.9 ± 1.3	3.0 ± 1.4	0.416
Homework time (min/d)	109.3 ± 52.8	106.9 ± 54.4	0.371	106.0 ± 51.2	105.2 ± 53.9	0.860	110.9 ± 53.6	108.4 ± 54.8	0.455
Screen time ≤ 2 h/d (%)	295 (87.8)	11,997 (87.1)	0.706	101 (91.0)	5804 (88.8)	0.467	194 (86.2)	6193 (85.6)	0.781
Screen time (min/d)	61.5 ± 59.3	66.6 ± 57.7	0.112	54.7 ± 55.6	61.6 ± 54.9	0.184	64.9 ± 60.9	71.1 ± 59.8	0.128
Television (min/d)	37.3 ± 37.4	39.8 ± 37.3	0.201	36.8 ± 37.2	38.4 ± 36.8	0.613	37.6 ± 37.6	41.0 ± 37.7	0.154
Computer (min/d)	25.5 ± 34.0	27.6 ± 33.3	0.259	19.6 ± 29.8	23.9 ± 30.3	0.132	28.3 ± 35.5	30.8 ± 35.5	0.279
**Sleeping**									
Night sleep latency (min/d)	18.2 ± 16.3	16.8 ± 12.9	0.074	17.9 ± 15	16.0 ± 12.4	0.083	18.4 ± 17	17.4 ± 13.4	0.367
Total sleep duration (h/d)	9.6 ± 0.9	9.7 ± 1.0	0.024	9.6 ± 0.8	9.7 ± 0.9	0.421	9.6 ± 0.9	9.7 ± 1.0	0.034
Night sleep duration (h/d)	8.9 ± 0.8	8.9 ± 0.8	0.094	8.9 ± 0.7	8.9 ± 0.8	0.646	8.8 ± 0.8	9.0 ± 0.8	0.011
Night sleep ≥ 9 h/d (%)	165 (40.6)	7383 (46.4)	0.021	57 (43.5)	3367 (44.3)	0.852	108 (39.3)	4016 (48.4)	0.003
Afternoon sleep duration (min/d)	43.1 ± 31.5	45.2 ± 31.4	0.195	42.2 ± 32.4	47.3 ± 30.4	0.060	43.6 ± 31.1	43.3 ± 32.2	0.879

MVPA, moderate-to-vigorous physical activity; VPA, vigorous physical activity; MPA, moderate physical activity. Note: * Differences of continuous and categorical variables between children with and without physician-diagnosed asthma were evaluated using independent *t*-tests and Chi-square tests, respectively.

**Table 3:** Analysis of the association between physician-diagnosed asthma and weight status stratified by gender, age, and lifestyle factors.

Physician-Diagnosed Asthma ^†^	Underweight ^#^		Overweight ^#^		Obesity ^#^	
	*OR* (95% *CI*)	*P*_interaction_	*OR* (95% *CI*)	*P*_interaction_	*OR* (95% *CI*)	*P*_interaction_
Overall	0.68 (0.35, 1.29)		1.18 (0.83, 1.68)		1.51 (1.03, 2.21) *	
Gender		0.807		0.925		0.178
Boys	0.71 (0.31, 1.65)		1.24 (0.80, 1.90)		1.69 (1.12, 2.55) *	
Girls	0.62 (0.22, 1.71)		1.13 (0.60, 2.11)		0.63 (0.15, 2.62)	
Age group		<0.001		<0.001		<0.001
6~8 years	1.32 (0.63, 2.78)		1.12 (0.62, 2.00)		1.36 (0.71, 2.62)	
9~12 years	0.23 (0.06, 0.92) *		1.22 (0.78, 1.90)		1.57 (0.97, 2.54)	
MVPA		0.294		0.271		0.003
≥60 min/d	0.46 (0.11, 1.90)		0.83 (0.42, 1.67)		1.03 (0.49, 2.15)	
<60 min/d	0.76 (0.37, 1.57)		1.36 (0.90, 2.05)		1.73 (1.10, 2.73) *	
Screen time		0.209		0.012		0.014
≤2 h/d	0.58 (0.28, 1.19)		0.99 (0.67, 1.47)		1.25 (0.81, 1.92)	
>2 h/d	1.93 (0.42, 8.93)		3.92 (1.56, 9.88) *		4.79 (1.83, 12.54)	
Night sleep duration		0.115		0.084		0.015
≥9 h/d	1.12 (0.48, 2.63)		1.66 (0.96, 2.87)		1.88 (0.98, 3.62)	
<9 h/d	0.41 (0.15, 1.14)		0.96 (0.60, 1.53)		1.31 (0.81, 2.11)	

MVPA, moderate to vigorous physical activity. Values are expressed as odds ratio (*OR*) and 95% confidence interval (*CI*) derived from the nominal logistic regression analysis. All models were adjusted for gender, age group, paternal education level, maternal education level, monthly family income, MVPA level, screen time level, night sleep duration level, and food intake, except when stratified. Note: ^#^ Compared to normal weight. ^†^ Compared to children without physician-diagnosed asthma. * *p* < 0.05.

**Table 4:** Lifestyle factors as mediators in the association between physician-diagnosed asthma and weight status.

Mediator	Effect	Underweight ^#^	Overweight ^#^	Obesity ^#^
MVPA	Total	0.70 (0.40, 1.21)	1.28 (0.95, 1.72)	1.48 (1.06, 2.06) *
	Direct	0.70 (0.40, 1.21)	1.28 (0.95, 1.72)	1.48 (1.06, 2.06) *
	Indirect	1.00 (0.99, 1.01)	1.00 (1.00, 1.00)	1.00 (1.00, 1.00)
Screen time	Total	0.65 (0.36, 1.17)	1.30 (0.96, 1.76)	1.47 (1.04, 2.06) *
	Direct	0.65 (0.36, 1.17)	1.30 (0.96, 1.76)	1.47 (1.04, 2.06) *
	Indirect	1.00 (1.00, 1.01)	1.00 (1.00, 1.01)	1.00 (1.00, 1.01)
Sleeping	Total	0.73 (0.44, 1.21)	1.36 (1.04, 1.79) *	1.51 (1.11, 2.06) *
	Direct	0.72 (0.43, 1.20)	1.37 (1.04, 1.80) *	1.53 (1.12, 2.08) *
	Indirect	1.00 (0.97, 1.01)	1.01 (1.00, 1.02)	1.01 (0.99, 1.03)

MVPA, moderate-to-vigorous physical activity; All models were adjusted for gender, age, paternal education level, maternal education level, and monthly family income. Note: ^#^ Compared with normal weight. * *p* < 0.05.
